# The clinical features and prognoses of anti-MDA5 and anti-aminoacyl-tRNA synthetase antibody double-positive dermatomyositis patients

**DOI:** 10.3389/fimmu.2022.987841

**Published:** 2022-08-30

**Authors:** Xixia Chen, Lu Zhang, Qiwen Jin, Xin Lu, Jieping Lei, Qinglin Peng, Guochun Wang, Yongpeng Ge

**Affiliations:** ^1^ Peking University, China-Japan Friendship School of clinical medicine, Beijing, China; ^2^ Department of Rheumatology, China-Japan Friendship Hospital, Beijing, China; ^3^ Data and Project Management Unit, Institute of Clinical Medical Sciences, China-Japan Friendship Hospital, Beijing, China; ^4^ Department of Rheumatology, Beijing Key Lab for Immune-Mediated Inflammatory Diseases, China-Japan Friendship Hospital, Beijing, China

**Keywords:** dermatomyositis, anti-MDA5 antibody, anti-aminoacyl-tRNA synthetase, myositis-specific auto-antibody, interstitial lung disease

## Abstract

**Objective:**

To explore the clinical features and prognoses of dermatomyositis (DM) associated with a double-positive anti-MDA5 and anti-aminoacyl-tRNA synthetase (anti-ARS) antibody presentation.

**Methods:**

We retrospectively analyzed 1280 consecutive patients with idiopathic inflammatory myopathy (IIM). Individuals with anti-MDA5 and anti-ARS antibodies (anti-MDA5+/ARS+) were compared to anti-MDA5-/ARS+ and anti-MDA5+/ARS- control individuals based on clinical, pulmonary radiological characteristics, treatment, and follow-up information.

**Results:**

Six individuals (0.47%) presented with anti-MDA5+/ARS+; of these, 2 (33.3%) were anti-PL-12+, 2 (33.3%) were anti-Jo-1+, 1 (16.7%) was anti-EJ+, and 1 (16.7%) was anti-PL-7+. Hallmark cutaneous manifestations, including Gottron’s sign (100%), heliotrope rash (50%), mechanic’s hand (66.7%), and skin ulcers (16.7%) were common. Anti-MDA5+/ARS+ patients tended to have higher ferritin levels (p = 0.038) than anti-MDA5-/ARS+ group, and higher CD4+ T-cell counts (p = 0.032) compared to the anti-MDA5+/ARS- group. Radiologically, NSIP with OP overlap was predominant (60%). Consolidation (60%), ground-glass attenuation (GGA) (80%), traction bronchiectasis (80%), and intralobular reticulation (100%) were common in anti-MDA5+/ARS+ individuals. All were diagnosed with ILD and 50% were categorized as RPILD. All patients received glucocorticoids combined with one or more immunosuppressants. Most (83.3%) had a good prognosis following treatment, but there was no difference in the survival rate between the three subgroups.

**Conclusion:**

Presentation with anti-MDA5+/ARS+ DM was rare. The clinical and radiological characteristics of anti-MDA5+/ARS+ DM combined the features of anti-MDA5+ and anti-ARS+ individuals. Individuals with anti-MDA5+/ARS+ antibodies may respond well to glucocorticoid therapy; glucocorticoids combined with one or more immunosuppressants may be considered a basic treatment approach.

## Introduction

Idiopathic inflammatory myopathies (IIM) are a heterogeneous group of autoimmune disorders usually characterized by chronic muscle inflammation with varying clinical manifestations, treatment responses, and prognoses. IIM can be classified into several subgroups: dermatomyositis (DM), anti-synthetase syndrome (ASS), immune-mediated necrotizing myopathy (IMNM), inclusion body myositis (IBM), polymyositis (PM), and overlap myositis (1). A major advance in the field of myositis was the discovery of auto-antibodies, called myositis-specific antibodies (MSA). As previous studies have reported (1, 2), MSAs are strongly associated with distinct clinical phenotypes and are therefore predictive of organ manifestations and potentially of prognosis.

ASS is characterized by the presence of unique anti-aminoacyl-tRNA synthetase (ARS) antibodies, which can be further sub-classified into: anti-histidyl (anti-Jo-1), anti-threonyl (anti-PL-7), anti-alanyl (anti-PL-12), anti-glycyl (anti-EJ), anti-isoleucyl (anti-OJ), etc (2). Anti-melanoma differentiation-associated gene 5 (MDA5) DM is a distinct subtype of DM. Patients with anti-MDA5 typically exhibit characteristic cutaneous manifestations, including palmar papules and deep ulcerations over joints, and have clinical amyopathic DM (CADM) with few muscular symptoms (3, 4). Anti-MDA5 DM is strongly associated with interstitial lung disease (ILD) in most regions and ethnicities, especially rapidly progressive ILD (RPILD) which has a poor clinical prognosis (5). ASS is a relatively homogeneous multisystem disease (6), characterized by fever, myositis, arthritis, mechanic’s hands, Raynaud’s phenomenon, and chronic relapsing ILD, and responds well to glucocorticoid and immunosuppressive agents. The coexistence of anti-MDA5 and anti-ARS antibodies is very rare; in fact, they are believed to be mutually exclusive (1). Very few such cases have been reported (7–11). In this study, we attempted to identify cases that are positive for anti-MDA5 and anti-ARS antibodies and explore the clinical features and prognosis of dermatomyositis in these individuals. This work will assist physicians in better understanding this disease and guide clinical decision-making.

## Methods

### Study design

We retrospectively analyzed the clinical data of 1280 consecutive patients with IIM hospitalized in the Department of Rheumatology at the China-Japan Friendship Hospital from January 2016 to September 2021. A diagnosis of IIM was based on the Bohan and Peter criteria (12) or 2004 European Neuromuscular Centre (ENMC) criteria (13). Patients with anti-MDA5 and anti-ARS antibodies (anti-MDA5+/ARS+) were enrolled. In addition, we selected controls by using a randomly generated number table; these included 24 (1:4) ARS antibody-positive patients without anti-MDA5 antibodies (anti-MDA5-/ARS+) and 24 (1:4) MDA5 antibody-positive patients without anti-ARS antibodies (anti-MDA5+/ARS-). The presence of ILD was evaluated *via* chest radiography or high-resolution computed tomography (HRCT). RPILD was defined as previously published (14). Patient demographic data, laboratory tests, therapy regimens, and follow-up information were captured and recorded in detail. In addition, we also conducted a literature review of the condition. The study protocol was approved by the Ethics Committee of the China-Japan Friendship Hospital (reference number: 2019-25-K19) and written informed consent was obtained from each participant. The study was conducted per the declaration of Helsinki, 2000.

### Clinical findings, laboratory parameters, and treatment

Patient background, clinical findings, and the treatment regimen were evaluated. Laboratory tests included lymphocyte count, creatine kinase (CK), lactic acid dehydrogenase (LDH), serum ferritin (FET), erythrocyte sedimentation rate (ESR), and C-reactive protein (CRP). Pulmonary function test (PFT) results [forced vital capacity (FVC) and diffusing capacity of the lungs for carbon monoxide (DLCO)] were also evaluated. In addition, we evaluated the overall HRCT score based on the classification by Ichikado (15). Patient treatment parameters including dosages of glucocorticoids (GC), steroid pulse therapy, immunosuppressive agents, intravenous immunoglobulin (IVIG), and biological agents were also recorded.

### Autoantibody detection

Anti-MDA5 and anti-ARS antibodies (antigens including Jo-1, PL-7, PL-12, EJ, and OJ) were quantified *via* immunoblotting according to the manufacturer’s instruction (EUROIMMUN, Lübeck, Germany). Myositis-associated antibodies (MAAs) (antigens including Ku, Ro-52, PM-Scl 100, and PM-Scl 75) were quantified in the same way. Sera that were positive (2+ or 3+) for anti-MDA5 and anti-ARS antibodies *via* immunoblotting were considered as positive. In order to avoid false positives, anti-synthetase ELISA Kit (MBL, Nagoya, Japan) was used to detect the presence of anti-ARS antibodies (including anti-Jo-1, anti-PL-7, anti-PL-12, anti-EJ and anti-KS antibodies) and anti-ARS antibodies’ level greater than 8.1 U/ml was considered as positive. Anti-MDA5 antibody was also detected using an ELISA Kit (MBL, Nagoya, USA) with a cut-off value of 32 U/ml (16).

### Prognosis and relapse

The follow-up period was calculated from the date of initial treatment to either the date of death or of the last investigation/evaluation. We evaluated patient prognoses and relapses during the follow-up period. Relapses were classified as ILD relapse, rash relapse, or myositis relapse. ILD relapse was recorded when all the following were present: aggravated respiratory status, deterioration in ILD based on radiological findings, and the need to institute treatment with GC or immunosuppressants. Rash and myositis relapses were recorded when a recurrence of rash or myositis necessitated the commencement of intensive treatment.

### Statistical analyses

All analyses were performed using IBM SPSS software (version 23.0, Armonk, NY, USA). Continuous data are presented as mean ± SD. Categorical variables are presented as frequencies and percentages. The continuous variables were compared *via* ANOVA with a Bonferroni post-hoc test when normally and homogeneous distributed, Kruskal Wallis H test with subsequent pairwise comparisons when not normally or inhomogeneous distributed. We used Fisher’s exact test to compare categorical variables and Chi-square with Bonferroni adjustment for *post hoc* tests. Survival among three groups was evaluated by applying the Kaplan-Meier (log-rank) test. All analyses were 2-tailed and p values <0.05 were considered to indicate statistical significance.

## Results

### Clinical characteristics of anti-MDA5+/ARS+ DM

We identified 6 individuals (0.47%) presenting as double positive for anti-MDA5 and anti-ARS antibodies. Of these, 2 (33.3%) were anti-PL-12+, 2 (33.3%) were anti-Jo-1+, 1 (16.7%) was anti-EJ+, and 1 (16.7%) was anti-PL-7+. A summary of the baseline clinical characteristics of the 6 double-positive patients is provided in **Table 1**. Comparisons between the clinical characteristics of the three subgroups (anti-MDA5+/ARS+, anti-MDA5-/ARS+, and anti-MDA5+/ARS-) are shown in **Table 2**.

**Table 1:** Characteristics of six anti-MDA5+/ARS+ dermatomyositis cases.

Case no.	1	2	3	4	5	6
General information						
Age at disease onset	46	51	51	53	37	53
Gender	F	F	M	M	F	M
Smoking history	–	–	–	+	–	–
Clinical manifestation						
Heliotrope rash	+	+	–	–	–	+
Gottron’s sign	+	+	+	+	+	+
Mechanic’s hand	+	+	+	–	+	+
Periungual erythema	–	–	+	–	–	–
Raynaud’s phenomenon	–	+	–	–	–	–
Skin ulcers	–	–	+	–	–	–
Arthritis	+	+	+	–	+	–
Fever	–	+	+	+	–	+
RPILD	–	+	–	+	–	+
Malignancy	–	–	–	–	–	–
Cardiac involvement	–	–	+	–	–	–
Laboratory investigations						
Anti-ARS antibodies	Anti-PL-12	Anti-PL-12	Anti-Jo-1	Anti-Jo-1	Anti-EJ	Anti-PL-7
MAAs	–	Ro-52	Ro-52	Ro-52	Ro-52	Ro-52
Lymphocyte count, (×10^9^/L)	0.65	0.90	0.87	2.87	1.74	0.86
CD4+ T-cell count, (×10^6^/L)	361	528	470	1398	814	805
Creatine kinase, (U/L)	174	28	540	283	126	23
LDH	371	269	329	1647	215	199
Max Ferritin, (ng/ml)	456.2	411.9	2171.0	>15000.0	33.4	1487.2
ESR, (mm/h)	23	34	12	54	8	36
CRP, (mg/dl)	1.24	2.51	0.19	1.64	0.45	<0.10
FVC, (%)	58.8	53.9	78.2	*	81.8	*
DLCO, (%)	28.5	34.2	76.0	*	44.6	*
Therapeutic regimen	MP pulse, PSL, CNI, IVIG	PSL, CNI, IVCY, IVIG	PSL, CNI, IVCY, IVIG	PSL, Tofacitinib, IVIG, VV-ECMO	PSL, CNI	PSL, CNI
Follow-up						
Follow-up period, (months)	55	45	37	1	26	12
ILD recurrence	–	–	–	–	–	–
Rash recurrence	–	–	+	–	+	–
Myositis recurrence	–	–	+	–	+	–
Outcome	alive	alive	alive	deceased	alive	alive

*: Patients were unable to complete the pulmonary function test due to severe dyspnea. +, present; -, absent.

F, female; M, male; MAAs, myositis-associated antibodies; LDH, lactate dehydrogenase; ESR, erythrocyte sedimentation rate; CRP, C-reactive protein; FVC, forced vital capacity; DLCO, diffusing capacity of the lungs for carbon monoxide; MP, methylprednisolone; PSL, prednisolone; CNI, calcineurin inhibitors; IVIG, intravenous immunoglobulin; IVCY, intravenous cyclophosphamide; VV-ECMO, venovenous extracorporeal membrane oxygenation.

**Table 2:** Comparison of clinical manifestations in different groups (anti-MDA5+/ARS+, anti-MDA5-/ARS+ and anti-MDA5+/ARS-) of IIM patients .

Parameter	Anti-MDA5+/ARS+ (N=6, group I)	Anti-MDA5-/ARS+ (N=24, group II)	Anti-MDA5+/ARS- (N=24, group III)	P value	P value
					Pair-wise comparison of the groups
**General information**					
Age, year	48.5 ± 6.2	52.2 ± 11.6	50.8 ± 10.6	0.542	–
Male, %	3 (50.0%)	6 (25.0%)	7 (29.2%)	0.529	–
Smoking history, %	1 (16.7%)	2 (8.3%)	4 (16.7%)	0.618	–
**Clinical manifestation**					
Heliotrope rash, %	3 (50.0%)	2 (8.3%)	18 (75.0%)	**<0.001**	0.041 (I-II) 0.329 (I-III) **<0.001 (II-III) ^#^**
Gottron’s sign, %	6 (100.0%)	9 (37.5%)	19 (79.2%)	**0.002**	**0.017 (I-II) ^#^** 0.553 (I-III) **0.003 (II-III) ^#^**
Mechanic’s hand, %	5 (83.3%)	11 (45.8%)	11 (45.8%)	0.280	–
Periungual erythema, %	1 (16.7%)	2 (8.3%)	6 (25.0%)	0.285	–
Raynaud’s phenomenon, %	1 (16.7%)	1 (4.2%)	0 (0.0%)	0.212	–
Skin ulcers, %	1 (16.7%)	2 (8.3%)	13 (54.2%)	**0.001**	0.501 (I-II) 0.175 (I-III) **0.001 (II-III) ^#^**
Arthritis, %	4 (66.7%)	7 (29.2%)	12 (50.0%)	0.170	–
Fever, %	4 (66.7%)	8 (33.3%)	11 (45.2%)	0.339	–
RPILD, %	3 (50.0%)	6 (25.0%)	11 (45.8%)	0.269	–
Malignancy, %	0 (0.0%)	0 (0.0%)	1 (4.2%)	1.000	–
Cardiac involvement, %	1 (16.7%)	3 (12.5%)	3 (12.5%)	1.000	–
**Laboratory investigation**					
Anti-Ro-52 antibody positive, %	5 (83.3%)	17 (70.8%)	17 (70.8%)	1.000	–
Lymphocyte count, (×10^9^/L)	0.89 (0.81, 2.02)	1.18 (0.76, 1.92)	0.68 (0.48, 0.98)	**0.003**	1.000 (I-II) 0.205 (I-III) **0.002 (II-III)**
CD4+ cell, (×10^6^/L)	666.5 (442.8, 960.0)	616.0 (390.0, 835.0)	261.0 (182.0, 417.0)	**0.002**	1.000 (I-II) **0.032 (I-III) 0.006 (II-III)**
Creatine kinase, (U/L)	150.0 (26.8, 347.3)	60.0 (35.0, 98.0)	70.0 (23.0, 147.0)	0.513	–
Ferritin, (ng/ml)	909.3 (131.5, 5378.3)	145.8 (42.6, 256.8)	551.6 (256.2, 1756.0)	**0.001**	0.083 (I-II) 1.000 (I-III) **0.001 (II-III)**
Max Ferritin, (ng/ml)	971.7 (317.3, 5378.3)	151.5 (57.4, 256.8)	1274.0 (414.3, 2026.1)	**<0.001**	0.038 (I-II) 1.000 (I-III) **<0.001 (II-III)**

^#^A Bonferroni-adjusted significance threshold (P < 0.017) for multiple comparison between three groups was used.

Statistically significant associations are shown in bold.

The anti-MDA5+/ARS+ group included 3 males and 3 females with a median age of 51 years (range 37–53). All showed the hallmark cutaneous manifestations of DM and presented with Gottron’s sign, 3 (50%) showed a Heliotrope rash, 1 (16.7%) showed periungual erythema and skin ulcers, 1 (16.7%) showed typical Raynaud’s phenomenon, 4 (66.7%) had arthritis, and 5 (83.3%) had mechanic’s hand. Compared to anti-MDA5-/ARS+, the anti-MDA5+/ARS+ group had a greater frequency of Heliotrope rash (p = 0.041) and Gottron’s sign (p = 0.017), but the between-group difference in Heliotrope rash was not significant after *post hoc* correction. All anti-MDA5+/ARS+ individuals were diagnosed with ILD and 3 (50%) were categorized as RPILD. Furthermore, none of them complicated with malignancy, while one patient (16.7%) presented with tachycardia without other manifestations of cardiac involvement.

In terms of laboratory investigations, five anti-MDA5+/ARS+ individuals (83.3%) were Ro-52 positive. At the initial visit, roughly half showed an elevated level of LDH, FET, ESR, CRP, and a decreased lymphocyte count. CD4+ T-cell counts in anti-MDA5+/ARS+ were higher than in anti-MDA5+/ARS- individuals (p = 0.032) but there was no difference when compared to anti-MDA5-/ARS+ (p = 1.000). Serum FET levels were elevated in anti-MDA5+/ARS+ compared to anti-MDA5-/ARS+ individuals (p = 0.038). PFT was performed in 4 anti-MDA5+/ARS+ individuals but could not be completed in the remaining 2 due to severe dyspnea. The former 4 showed a restrictive pattern and a decreased diffusing capacity.

### Radiological findings during anti-MDA5+/ARS+ DM

**Figure 1:**
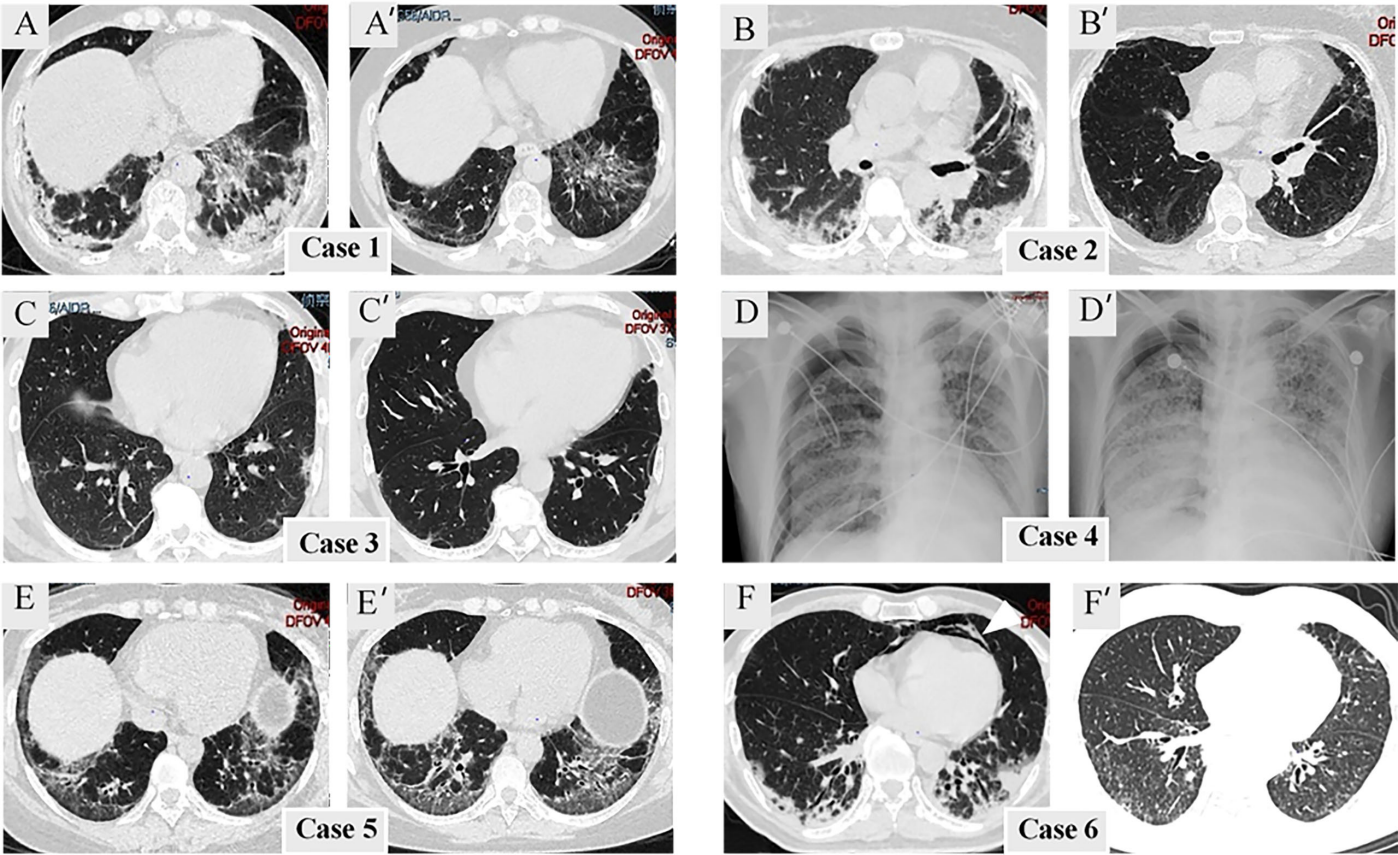
Radiological finding. **(A–F)** Radiological imaging at admission. **(A’–F’**) Radiological imaging following treatment. Mediastinal emphysema is indicated by the arrowheads.

One individual (Case 4) was unable to complete HRCT detection due to severe dyspnea and instead received bedside chest radiography. Others had HRCT on admission and during follow-up. **Supplementary Table S1** compares HRCT findings among the three subgroups (anti-MDA5+/ARS+, anti-MDA5-/ARS+, and anti-MDA5+/ARS-). Although there was no significant difference in HRCT findings between anti-MDA5+/ARS+ and the two control groups, we found that NSIP with OP overlap dominated (3/5, 60%), and most presented with consolidation in the subpleural area of the lung (**Figure 1**). The regions of consolidation were symmetrically distributed, mainly in both lower lobes. In addition, we observed ground-glass attenuation (GGA) in 4 (80%) individuals, reticulation in 5 (100%), and traction bronchiectasis in 4 (80%). Ichikado has suggested that an HRCT score > 230 is independently associated with a risk of death (15). In the present study, no patients with anti-MDA5+/ARS+ antibodies had an HRCT score >230. During the follow-up period, 3 individuals (Cases 1, 2, and 6) showed a remarkable improvement in ILD, while 2 showed no marked change (Cases 3 and 5).

### Treatment and follow-up

**Figure 2:**
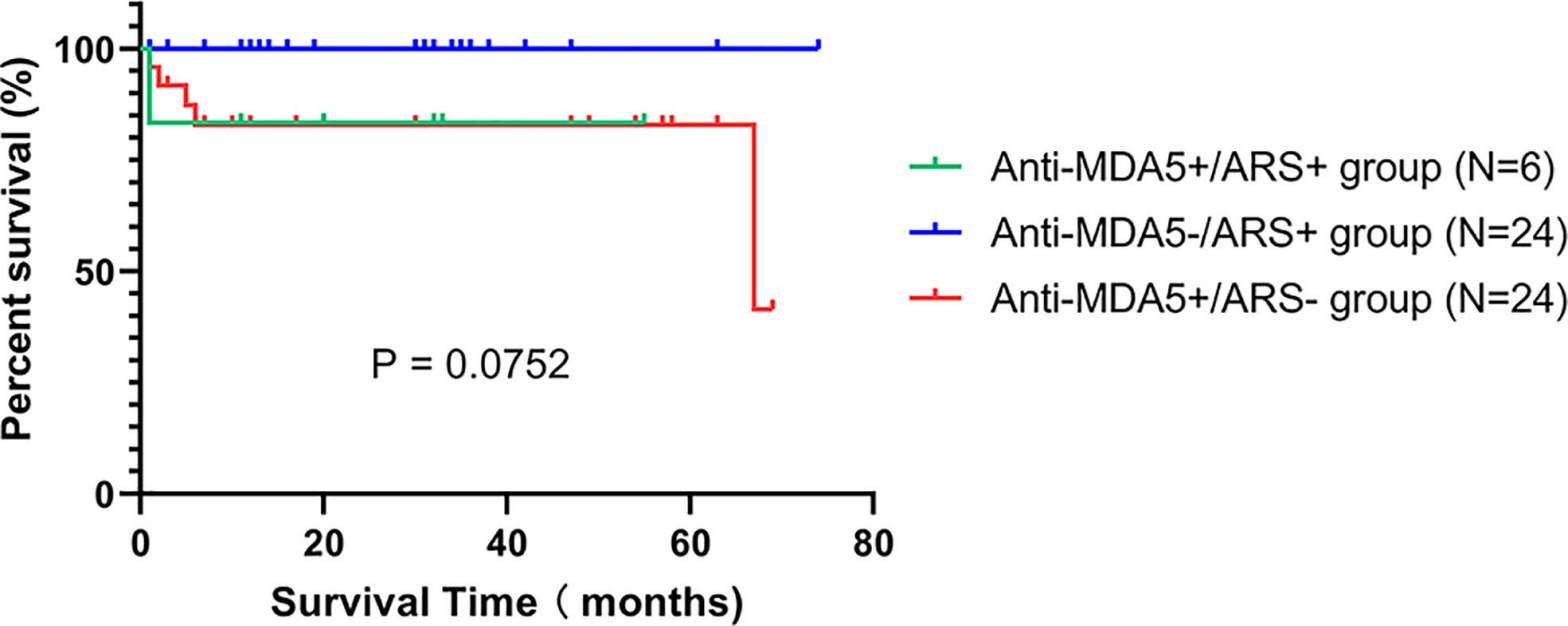
Kaplan-Meier curves for the anti-MDA5+/ARS+, anti-MDA5-/ARS+, and anti-MDA5+/ARS- subgroups.

All anti-MDA5+/ARS+ DM individuals received combination therapy (**Table 1**). At the initial visit, 1 received methylprednisolone pulse therapy (500mg×3d), while the others were treated with medium or high doses of prednisolone (PSL). Immunosuppressants were simultaneously administered; these included calcineurin inhibitors (CNI) (tacrolimus or cyclosporin A), intravenous cyclophosphamide (IVCY), or tofacitinib. When comparing the treatment regimens among the three subgroups (**Supplementary Table S2**), we found that compared to anti-MDA5-/ARS+, anti-MDA5+/ARS+ individuals showed a higher tendency to require triple therapy (PSL, CNI, and IVCY; p = 0.034) and IVIG (p = 0.007), although a significant difference was not observed on triple therapy after *post hoc* correction. Following treatment, 5/6 (83.3%) experienced relief of respiratory symptoms and showed a good prognosis except for 1 failure of intensive treatment resulting in death (Case 4). During the follow-up period, 2 (33.3%) experienced rash and myositis recurrence, while none had a recurrence of ILD. Follow-up FET, CK, lymphocyte counts, and HRCT scores improved over time (**Supplementary Figure S1**). There was no difference in the overall survival rate between the three subgroups (p > 0.05) (**Figure 2**).

### Complications during treatment

Compared with anti-MDA5-/ARS+, infection was more common in anti-MDA5+/ARS+ individuals (**Supplementary Table S3**). The clinical course was complicated in 66.7% of the anti-MDA5+/ARS+ individuals by the presence of at least one pathogen. We identified bacterial infection in 2 (33.3%), fungal infection in 2 (33.3%), and cytomegalovirus (CMV) in 1 (16.7%) of the cases. The infections were well controlled with intensive treatment in most of these cases. Mediastinal emphysema is a frequent complication of anti-MDA5+ DM (17) and was observed in 1/6 (16.7%) cases in this study.

### Literature review

We analyzed five published case reports (7–11). Their characteristics are summarized in **Table 3**. Together with our cohort, 11 anti-MDA5+/ARS+ were identified, including 3 adult males and 8 adult females with a median age of 51 years (range 27–53). 10 (90.9%) were Asian. The main anti-ARS antibodies were anti-PL-12 (4/11, 36.3%), anti-PL-7 (3/11, 27.3%), anti-Jo-1 (2/11, 18.2%), and anti-EJ (2/11, 18.2%). Cutaneous lesions (100%) and ILD (100%) were the most common reported symptoms. 7/11 (63.6%) presented with RPILD and anti-Ro-52 antibodies were detected in 75% (6/8) of cases. Radiologically, consolidations (6/10, 70%) and GGA (9/10, 90%) with a peripheral distribution were prevalent. Traction bronchiectasis (5/10, 50%) and intralobular reticulation (8/10, 80%) were also frequently seen. All cases were treated with glucocorticoids and immunosuppressants. Some had additional treatments, such as IVIG, rituximab, plasmapheresis, and VV-ECMO. Following treatment, 9 (81.8%) recovered and 2 (18.2%) failed intensive treatment and died of respiratory failure. During follow-up, 3/9 (33.3%) experienced a recurrence of ILD, 2 (22.2%) of rash, and 3 (33.3%) of myositis.

**Table 3:** Characteristics of the five published anti-MDA5+/ARS+ dermatomyositis cases.

Ref.	Naniwa T (7)	Takeuchi Y (8)	Li ZY (9)	Hama S (10)	Hiramatsu T (11)
Age/Gender	43/Female	53/Female	27/Female	51/Female	32/Female
Race	Japanese	Japanese	Hispanic	Japanese	Japanese
Anti-ARS antibody	Anti-PL-7	Anti-EJ	Anti-PL-7	Anti-PL-12	Anti-PL-12
MAAs	Anti-SSA	ND	ND	ND	Anti-Ro-52, anti-CCP
Skin manifestations	Heliotrope rash, facial erythema, shawl sign, Gottron’s papules, periungual erythema, nail fold bleeding	Heliotrope rash, facial erythema, Gottron’s papules with ulcers, mechanic’s hands, periungual erythema	Gottron’s papules	Heliotrope rash, Gottron’s papules with ulcers, mechanic’s hands	Facial erythema, Gottron’s papules, mechanic’s hands, palmar papules
Chest CT findings	Consolidations and GGA with peripheral distribution, subpleural line, intralobular reticular opacities	Initial: Lower peripheral reticulation and GGA. Exacerbation: Newly developed random GGA	Extensive GGA bilaterally without bronchiectasis	Upper random GGA, lower peripheral reticulation with consolidation and traction bronchiectasis	Subpleural consolidation and GGA mainly in the bilateral lower lobes
RPILD	+	+	+	+	–
FET, (ng/ml)	95.1	ND	ND	696	50
LDH, (U/L)	370	ND	ND	ND	276
CK, (U/L)	1078	Elevation	Normal	45	274
Treatment	PSL + TAC + IVCY + IVIG	PSL + TAC + IVCY + Plasmapheresis	MP pulse + IVIG + RTX+ VV-ECMO	MP pulse + TAC + IVCY + IVIG	PSL + TAC + IVCY
Follow up period	3.5 years	15 years	33 days	6 months	6 months
Recurrence	+ (ILD and myositis)	+ (ILD)	–	–	+ (ILD)
Outcome	Alive	Alive	Deceased	Alive	Alive

+, present; -, absent.

MAAs, myositis-associated antibodies; GGA, ground-glass attenuation; FET, ferritin; LDH, lactate dehydrogenase; CK, creatine kinase; PSL, prednisolone; TAC, tacrolimus; IVCY, intravenous cyclophosphamide; IVIG, intravenous immunoglobulin; MP, methylprednisolone; RTX, rituximab; VV-ECMO, venovenous extracorporeal membrane oxygenation; ND, not described.

## Discussion

In the present study, we identified and reviewed six anti-MDA5+/ARS+ DM cases. Our analyses raised three important clinical issues. First, anti-MDA5+/ARS+ DM shows clinical characteristics that combine the features of anti-MDA5+ DM and anti-ARS+ DM. Second, NSIP with OP overlap is predominant in anti-MDA5+/ARS+ associated ILD. Third, anti-MDA5+/ARS+ patients appear to respond to glucocorticoid therapy and glucocorticoid combined with one or more immunosuppressants; as such, this approach should be considered in cases of anti-MDA5+/ARS+ DM.

A clinical presentation with combined anti-MDA5 and anti-ARS antibodies in DM is very rare. As such we know little about the characteristics of this disease subgroup. To our knowledge, the prevalence of anti-MDA5+/ARS+ in IIM patients is approximately 0.47%. In the present study, we found that cutaneous lesions and ILD were the most common symptoms in anti-MDA5+/ARS+ DM. Moreover, we found that periungual erythema, skin ulceration (characteristic of anti-MDA5+ DM), and mechanic’s hands (characteristic of anti-ARS+ DM) were common in anti-MDA5+/ARS+ DM (18). Previous studies reported that the prevalence of ILD in anti-MDA5+ DM ranges from 50 to 100% (19, 20) and that anti-MDA5-associated RPILD is more common than anti-ARS-associated RPILD (21). In our cohort, all anti-MDA5+/ARS+ patients were diagnosed with ILD and 50% were categorized as RPILD. Together with the published cases, 63.6% of the double-positive individuals presented with RPILD, which is more closely aligned with anti-MDA5+ DM (22). However, Yuhui Li et al. (21, 23) reported mortality rates of 30–40% in anti-MDA5 antibody-associated RPILD, which is higher than the mortaility rate of anti-MDA5+/ARS+ associated RPILD in our cohort (28.6%). Therefore, the clinical course of anti-MDA5+/ARS+ may be better and more closely resembled that of anti-ARS+ DM.

To date, numerous biomarkers have been reported to be associated with DM-ILD (15, 24). Ferritin is a well-characterized serum biomarker for anti-MDA5+ DM-ILD and correlates with the severity and prognosis of ILD; reported cutoff values vary from 500 to 1500 ng/ml (25). We showed that the maximum ferritin levels of anti-MDA5+/ARS+ DM were significantly higher than in anti-MDA5-/ARS+ DM but not different from anti-MDA5+/ARS- DM. Notably, one patient in our cohort died of respiratory failure with extremely high levels of ferritin (> 15,000.0 ng/mL). Systemic macrophage activation, which is correlated with hyperferritinemia, is potentially related to the pathogenesis of anti-MDA5 antibody-associated RPILD (26, 27). Whether macrophage activation is also involved in the pathophysiology of anti-MDA5+/ARS+ DM and directly modulates ferritin levels remains to be tested. Apart from hyperferritinemia, lymphocytopenia is also reported as a risk factor in anti-MDA5+ DM. There are several possible explanations for the decrease of lymphocytes in DM. Shu et al. found that DM is associated with suppressed CD3+ T-cell autophagy, leading to increased T-lymphocyte apoptosis and lymphocytopenia (28). Kamphuis E et al. demonstrated that increased type 1 interferons can downregulate central memory and naïve T lymphocytes, activate B lymphocytes, and eventually result in lymphocytopenia (29). We found that CD4+ T-cell counts were higher in anti-MDA5+/ARS+ DM than anti-MDA5+/ARS- DM patients, implying a favorable prognostic immune state in the former group. Based on these characteristics of anti-MDA5+/ARS+ DM, we concluded that anti-MDA5+/ARS+ DM patients show a combined presentation of anti-MDA5+ DM and anti-ARS+ DM; we investigated this hypothesis further through radiological analyses.

Using HRCT, most anti-MDA5+/ARS+ patients (60%) were diagnosed as having NSIP with OP overlap and two (40%) had NSIP. Consolidation, GGA, traction bronchiectasis, and intralobular reticulation were frequently observed in these patients. As described in previous studies (25), lower consolidation/GGA patterns and random GGA patterns are mainly found in anti-MDA5+ DM, whereas a lower reticulation pattern is the main finding among anti-MDA5- DM. The latter HRCT pattern corresponds with NSIP and likely with anti-ARS+ DM patients. Therefore, we thought that the radiological characteristics of anti-MDA5+/ARS+ DM also combined the features of anti-MDA5+ DM and anti-ARS+ DM. Although some studies suggested that NSIP with OP overlap may correlate with the acute or subacute form of onset of ILD and become resistant to therapy and progress to fibrosis (30), most anti-MDA5+/ARS+ patients in our cohort showed a remarkable improvement or remained stable following treatment.

In terms of treatment, there is no generally accepted therapy guideline for MSA antibody-positive or double-antibody positive cases. Based on the conventional treatment of anti-MDA5+ DM, glucocorticoid monotherapy may be inadequate, and more intensive therapy might improve prognosis (31). Together with published case reports, all identified anti-MDA5+/ARS+ DM cases received glucocorticoids and additional treatments, including CNI, IVCY, IVIG, tofacitinib, rituximab, plasmapheresis, and VV-ECMO. Most responded to these treatments and showed good prognosis, except for two who died of respiratory failure despite intensive immunosuppressive therapy. Hiramatsu T (11) reported on a female case with anti-MDA5+/ARS+ DM, who was initially refractory to glucocorticoids combined with tacrolimus; her condition was subsequently stabilized by IVCY. Therefore, glucocorticoids combined with one or more immunosuppressants may offer a basic treatment approach in cases of anti-MDA5+/ARS+ DM. In our cohort, IVIG was more commonly used in anti-MDA5+/ARS+ DM compared to anti-MDA5-/ARS+ DM. Its value arises from the inhibition of cytokine production and enhancement of the clearance of pathogenic autoantibodies (32). Thus, IVIG can also be considered in these patients. Furthermore, plasma exchange, VV-ECMO, and lung transplantation might be considered in life-threatening situations although their efficacy remain controversial (33, 34).

In our cohort, two individuals (33.3%) experienced a recurrence of rash and myositis but none had a recurrence of ILD during the follow-up. In our literature review, we identified three of five cases that presented with an ILD recurrence; one of these also had a recurrence of myositis. How disease recurrence factors into the clinical course of anti-MDA5+/ARS+ DM is still unknown. In our survival analyses, there was no significant difference between the three subgroups, which partly stems from the unavoidably small sample size. Thus, the prognosis of anti-MDA5+/ARS+ DM remains uncertain and warrants further investigation. Additionally, we observed infection and mediastinal emphysema in anti-MDA5+/ARS+ DM. Many studies have shown that infection and mediastinal emphysema can increase the risk of death in IIM (17, 35, 36). Therefore, clinicians also need to be highly vigilant of these complications in anti-MDA5+/ARS+ DM.

This study presents critically important data but we recognize some limitations. First, this was a small, retrospective study conducted at a single institution. Second, the small sample size limited statistical power. Although anti-MDA5+/ARS+ DM is very rare, we believe that as this and other studies are published, we can develop an increasingly precise understanding of this condition and its most effective therapies.

In summary, anti-MDA5+/ARS+ DM is very rare. The clinical and radiological characteristics of anti-MDA5+/ARS+ DM show combined features of anti-MDA5+ DM and anti-ARS+ DM. Anti-MDA5+/ARS+ patients might respond well to glucocorticoid therapy and glucocorticoid combined with one or more immunosuppressants. Further studies and meta-analyses will gradually help us to improve the precision of these findings.

## Data availability statement

The original contributions presented in the study are included in the article/**Supplementary Material**. Further inquiries can be directed to the corresponding author.

## Ethics statement

Written informed consent was obtained from the individual(s) for the publication of any potentially identifiable images or data included in this article.

## Author contributions

YG conceived of the review and supervised the project. XC wrote the manuscript. LZ, XL and QJ contributed to literature review and editing. JL and QP contributed to literature review and compiled figures. GW contributed to content and editing. All authors contributed to the article and approved the submitted version.

## Funding

This work was supported by the National High Level Hospital Clinical Research Funding [2022-NHLHCRF-YS-02]; and National Natural Science Foundation of China [81971521, 82171788].

## Acknowledgments

The authors would like to express their gratitude to EditSprings (https://www.editsprings.cn) for the expert linguistic services provided.

## Conflict of interest

The authors declare that the research was conducted in the absence of any commercial or financial relationships that could be construed as a potential conflict of interest

## Publisher’s note

All claims expressed in this article are solely those of the authors and do not necessarily represent those of their affiliated organizations, or those of the publisher, the editors and the reviewers. Any product that may be evaluated in this article, or claim that may be made by its manufacturer, is not guaranteed or endorsed by the publisher.
