# Supplementary material for: The clinical features and prognoses of anti-MDA5 and anti-aminoacyl-tRNA synthetase antibody double-positive dermatomyositis patients
Source: Front Immunol. 2022 Aug 30;13:987841. doi: 10.3389/fimmu.2022.987841 (PMC9468482; doi:10.3389/fimmu.2022.987841)

Supplementary Material

# Supplementary Tables

**Table S1.** Radiological analyses in different groups (anti-MDA5+/ARS+, anti-MDA5-/ARS+ and anti-MDA5+/ARS-) of IIM patients

| Variable | Anti-MDA5+/ARS+ (N=5, group I) | Anti-MDA5-/ARS+ (N=24, group II) | Anti-MDA5+/ARS- (N=24, group III) | P value | P value  Pair-wise comparison of the group |
| --- | --- | --- | --- | --- | --- |
| **Radiological diagnosis** |  |  |  |  |  |
| NSIP, n (%) | 2 (40.0%) | 18 (75.0%) | 11 (45.8%) | 0.080 | 0.287 (I-II)  1.000 (I-III)  0.039 (II-III) |
| OP, n (%) | 0 (0.0%) | 1 (4.2%) | 7 (29.2%) | **0.048** | 1.000 (I-II)  0.296 (I-III)  0.048 (II-III) |
| UIP, n (%) | 0 (0.0%) | 2 (8.3%) | 1 (4.2%) | 1.000 | - |
| NSIP with OP overlap, n (%) | 3 (60.0%) | 3 (12.5%) | 5 (20.8%) | 0.060 | 0.046 (I-II)  0.112 (I-III)  0.701 (II-III) |
| **HRCT findings** |  |  |  |  |  |
| Ground glass attenuation, n (%) | 4 (80.0%) | 21 (87.5%) | 19 (79.2%) | 0.669 | - |
| Consolidation, n (%) | 3 (60.0%) | 5 (20.8%) | 17 (70.8%) | **0.001** | 0.112 (I-II)  0.663 (I-III)  **0.001 (II-III)** ^#^ |
| Reticulation, n (%) | 5 (100.0%) | 23 (95.8%) | 22 (91.7%) | 1.000 | - |
| Traction bronchiectasis, n (%) | 4 (80.0%) | 18 (75.0%) | 7 (29.2%) | **0.002** | 1.000 (I-II)  0.054 (I-III)  **0.001 (II-III)** ^#^ |
| Honeycombing, n (%) | 0 (0.0%) | 2 (8.3%) | 2 (8.3%) | 1.000 | - |
| Lower lung volume loss, n (%) | 3 (60.0%) | 18 (75.0%) | 14 (58.3%) | 0.522 | - |
| Emphysema, n (%) | 0 (0.0%) | 0 (0.0%) | 2 (8.3%) | 0.582 | - |
| Cyst, n (%) | 0 (0.0%) | 1 (4.2%) | 1 (4.2%) | 1.000 | - |
| Swollen mediastinal lymph nodes, n (%) | 0 (0.0%) | 7 (29.2%) | 6 (25.0%) | 0.582 | - |
| Pleural irregularities and thickening, n (%) | 4 (80.0%) | 14 (58.3%) | 12 (50.0%) | 0.513 | - |
| Lower predominant, n (%) | 5 (100.0%) | 18 (75.0%) | 15 (62.5%) | 0.297 | - |
| **HRCT score, %** | 167.0 (117.0, 194.0) | 146.5 (116.3, 190.3) | 130.5 (113.0, 162.8) | 0.399 | - |

#: A Bonferroni-adjusted significance threshold (P < 0.017) for multiple comparison between three groups was used.

Statistically significant associations are shown in bold.

**Table S2.** Comparison of treatment in different groups (anti-MDA5+/ARS+, anti-MDA5-/ARS+ and anti-MDA5+/ARS-) of IIM patients

|  | Anti-MDA5+/ARS+ (N=6, group I) | Anti-MDA5-/ARS+ (N=24, group II) | Anti-MDA5+/ARS- (N=24, group III) | P value | P value  Pair-wise comparison of the groups |
| --- | --- | --- | --- | --- | --- |
| PSL alone | 0 (0.0%) | 2 (8.3%) | 6 (25.0%) | 0.199 | - |
| PSL + CNI | 3 (50.0%) | 5 (20.8%) | 9 (37.5%) | 0.287 | - |
| PSL + Others* | 1 (16.7%) | 17 (70.8%) | 4 (16.7%) | **<0.001** | - |
| PSL + CNI + IVCY | 2 (33.3%) | 0 (0.0%) | 5 (20.8%) | **0.015** | 0.034 (I-II)  0.603 (I-III)  0.050 (II-III) |
| Add on RTX | 0 (0.0%) | 0 (0.0%) | 3 (12.5%) | 0.327 | - |
| Add on IVIG | 4 (66.7%) | 2 (8.3%) | 15 (62.5%) | **<0.001** | **0.007 (I-II)** ^#^  1.000 (I-III)  **<0.001 (II-III)** ^#^ |

*: Others including intravenous cyclophosphamide, tofacitinib, baricitinib, methotrexate and hydroxychloroquine. PSL, prednisolone; CNI, calcineurin inhibitors; IVCY, intravenous cyclophosphamide; RTX, retuximab; IVIG, intravenous immunoglobulin.

#: A Bonferroni-adjusted significance threshold (P < 0.017) for multiple comparison between three groups was used.

Statistically significant associations are shown in bold.

**Table S3. Complications in anti-MDA5+/ARS+, anti-MDA5-/ARS+, and anti-MDA5+/ARS- IIM**

|  | Anti-MDA5+/ARS+ (N=6, group I) | Anti-MDA5-/ARS+ (N=24, group II) | Anti-MDA5+/ARS- (N=24, group III) | P value | P value  Pair-wise comparison of the groups ^#^ |
| --- | --- | --- | --- | --- | --- |
| Infection, n (%) | 4 (66.7%) | 6 (25.0%) | 15 (62.5%) | **0.022** | 0.141 (I-II)  1.000 (I-III)  **0.009 (II-III)** |
| Bacterial, n (%) | 2 (33.3%) | 3 (12.5%) | 7 (29.2%) | 0.329 | - |
| Fungal, n (%) | 2 (33.3%) | 1 (4.2%) | 9 (37.5%) | **0.009** | 0.094 (I-II)  1.000 (I-III)  **0.004 (II-III)** |
| CMV, n (%) | 1 (16.7%) | 2 (8.3%) | 4 (16.7%) | 0.618 | - |
| Mediastinal emphysema, n (%) | 1 (16.7%) | 0 (0.0%) | 5 (20.8%) | 0.057 | - |

CMV, cytomegalovirus.

#: A Bonferroni-adjusted significance threshold (P < 0.017) for multiple comparison between three groups was used.

Statistically significant associations are shown in bold.

# Supplementary Figure


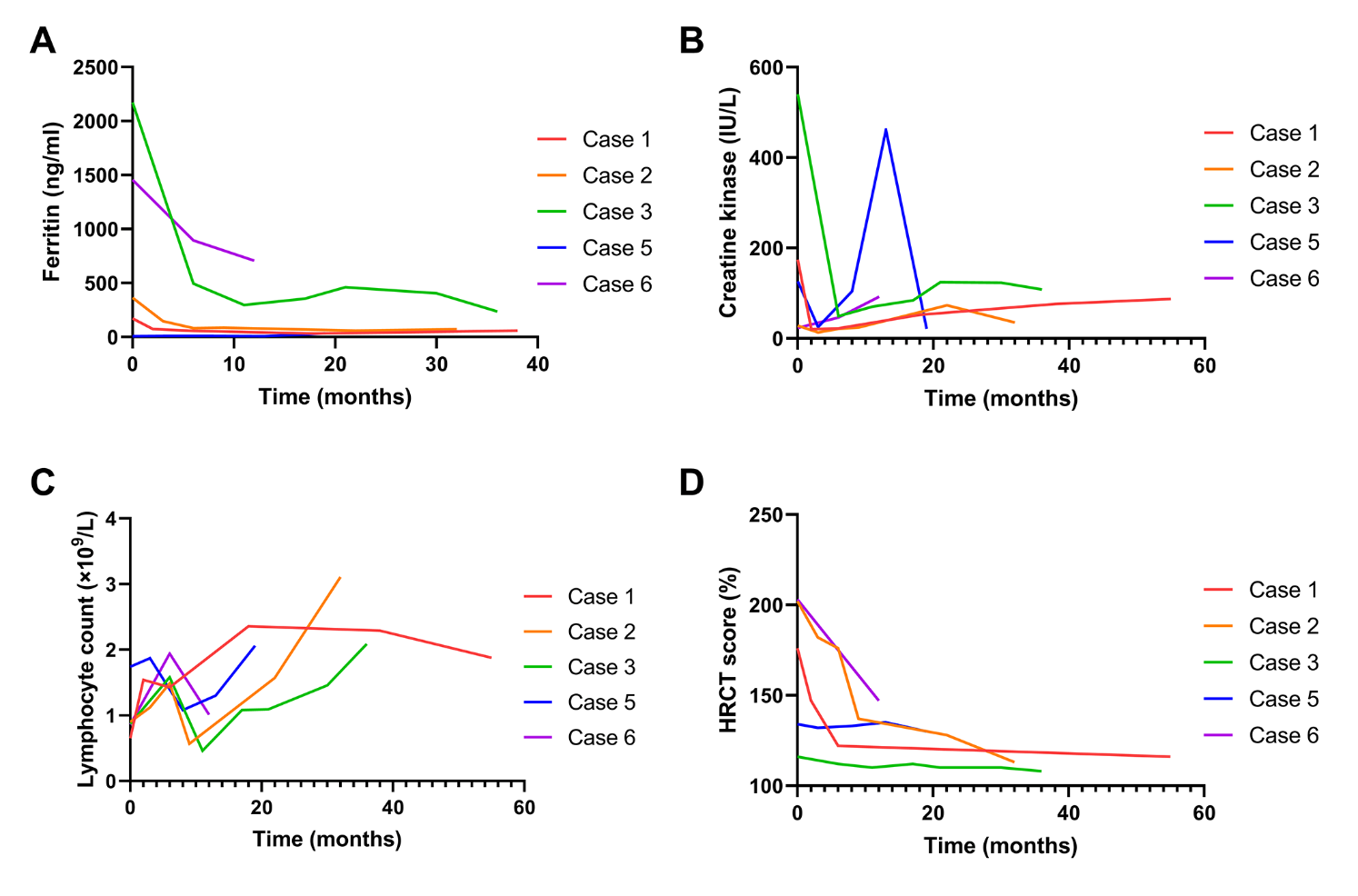


**Supplementary Figure S1.** Follow-up study of abnormal paraclinical factors in anti-MDA5+/ARS+ DM patients. Ferritin (A), creatine kinase (B), lymphocyte count (C) and HRCT score (D) showing improvement over time.


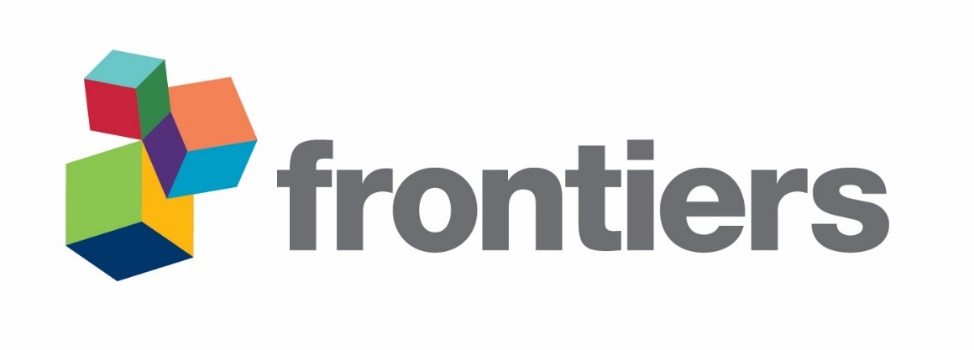

Supplement: Supplementary file 1 [file DataSheet_1.docx]
